# Supplementary material for: Mechanocaloric effects in superionic thin films from atomistic simulations
Source: Nat Commun. 2017 Oct 17;8:963. doi: 10.1038/s41467-017-01081-7 (PMC5645463; doi:10.1038/s41467-017-01081-7)
Supplement: Supplementary file 1 — Supplementary Information [file 41467_2017_1081_MOESM1_ESM.pdf]

SUPPLEMENTARY METHODS

The interatomic potentials adopted to investigate CaF_2 and Li_3OCl with molecular dynamics simulations are of the Born-Mayer-Huggins form:

$$V_{ij}(r) = A_{ij} e^{-\frac{r}{\rho_{ij}}} - \frac{C_{ij}}{r^6} + \frac{Z_i Z_j}{r} \ , \tag{1}$$

where subscripts i and j represent the ionic species in the system, r the radial distance between a couple of atoms, and the corresponding parameter values are reported in Supplementary Table I [1–3]. Each pairwise interatomic term is composed of three different contributions; the first one is of exponential type and accounts for the short-ranged atomic repulsion deriving from the overlapping between electron clouds; the second term is proportional to r^{-6} and represents the long-ranged atomic attraction due to dispersive van der Waals forces; the third term is the usual Coulomb interaction between puntual atomic charges.

The interatomic potential used to investigate AgI with molecular dynamics simulations is that due to Vashishta and Rahman [4], which is expressed as:

$$U_{ij}(r) = \frac{H_{ij}}{r^{n_{ij}}} - \frac{W_{ij}}{r^6} + \frac{Z_i Z_j}{r} - \frac{P_{ij}}{r^4} \ , \tag{2}$$

where again subscripts i and j represent the ionic present in the system, r the radial distance between a couple of atoms, and the corresponding parameter values are reported in Supplementary Table II. In this case, each pairwise interatomic term is composed of four different contributions; the first three terms in Eq. (2) are physically equivalent to those found in Eq. (1), with the only difference that the short-ranged repulsion between ions now is modelled with a negative power law; a fourth attractive contribution to the interaction energy is added that accounts for the effects of the electronic polarisabilities.

In order to accurately compute the thermodynamic shifts $\Delta S(\sigma, T)$ and $\Delta T(\sigma, T)$ in CaF_2, Li_3OCl and AgI thin films, we calculated the corresponding biaxial strains and heat capacities in a dense grid of (σ, T) points spaced by $\Delta\sigma = 0.1$ GPa and $\Delta T = 20$ K. Mechanical strains were estimated directly from the molecular dynamics simulations, and the heat capacity from the differences between the internal energies of simulations performed at T and $T \pm 20$ K conditions. Spline interpolations were subsequently applied to the calculated data points in order to obtain continuous and well-behaved $\epsilon(\sigma, T)$ and $\frac{\partial \epsilon}{\partial T}(\sigma, T)$ curves.

Supplementary Table I: Interatomic pairwise potential parameters used to describe CaF_2 [1,2] and Li_3OCl [3] in our classical molecular dynamics simulations. Note that $Z_{Ca} = +2$, $Z_F = -1$, $Z_{Li} = +1$, $Z_O = -2$, and $Z_{Cl} = -1$ in e units.

	A (eV)	ρ (Å)	C (eV Å^6)
		**CaF_2**	
$Ca^+ - F^-$	1717.441	0.287	0.102
$F^- - F^-$	2058.994	0.252	16.703
$Ca^+ - Ca^+$	0.0	0.0	0.0
		**Li_3OCl**	
$O^{2-} - O^{2-}$	22764.30	0.1490	13.185
$Cl^- - Cl^-$	5145.28	0.3066	20.523
$Li^+ - O^{2-}$	433.26	0.3138	0.0
$Li^+ - Cl^-$	421.04	0.3364	0.0
$Li^+ - Li^+$	360.53	0.1609	0.0

Supplementary Table II: Interatomic pairwise potential parameters used to describe AgI [4] in our classical molecular dynamics simulations.

**AgI**			
Z_{Ag}	+0.6	$H_{\mathrm{I-I}}$ (eV Å^7)	6427.1
Z_{I}	−0.6	$P_{\mathrm{Ag-Ag}}$ (eV Å^4)	0.0
$n_{\mathrm{Ag-Ag}}$	11	$P_{\mathrm{Ag-I}}$ (eV Å^4)	16.888
$n_{\mathrm{Ag-I}}$	9	$P_{\mathrm{I-I}}$ (eV Å^4)	33.776
$n_{\mathrm{I-I}}$	7	$W_{\mathrm{Ag-Ag}}$ (eV Å^6)	0.0
$H_{\mathrm{Ag-Ag}}$ (eV Å^{11})	0.21303	$W_{\mathrm{Ag-I}}$ (eV Å^6)	0.0
$H_{\mathrm{Ag-I}}$ (eV Å^9)	1647.4	$W_{\mathrm{I-I}}$ (eV Å^6)	99.767

The ΔS and ΔT values enclosed in Figs. 2-5 of the main text were obtained by numerically integrating those functions with respect to the applied biaxial stress, and considering $C_\sigma(\sigma, T) \approx C_\sigma(0, T)$.

SUPPLEMENTARY DISCUSSION

Ab initio molecular dynamics (AIMD) simulations based on density functional theory were performed in the canonical (N, V, T) ensemble for bulk CaF_2 (perfect and with vacancies), Li_3OCl (perfect and with vacancies), and AgI (perfect). The objective of these

calculations was to validate the reliability of the employed interatomic potential models in our molecular dynamics simulations.

The temperature in the AIMD simulations was kept fluctuating around a set-point value by using Nose-Hoover thermostats. Large simulation boxes containing up to 192 atoms were used in all the cases, and periodic boundary conditions were applied along the three corresponding Cartesian directions. Newton's equations of motion were integrated using the customary Verlet's algorithm and a time-step length of 10^{-3} ps. Γ-point sampling for integration within the first Brillouin zone was employed in all the AIMD simulations. Calculations comprised long simulation times of up to ~ 30 ps. We focused on the description of the superionic features, which consistently were identified through inspection of the mean squared displacement function obtained directly from the AIMD runs.

In two previous works [1,2], we have already validated the realiability of the CaF_2 interatomic potential employed in our molecular dynamics simulations involving perfect systems (i.e., with no vacancies). We have carried out further AIMD tests in order to check whether the same level of accuracy is maintained also for defective systems. In particular, we have performed classical and *ab initio* simulations for CaF_2 crystals considering a concentration of vacancies, c_v, of 5% and hydrostatic pressures of $P = 0$, 3, and 5 GPa. At those conditions, we obtain superionic transition temperatures of $T_s^{\rm DFT} = 900(100)$, 1075(100) and 1000(100) K with AIMD simulations, respectively, and of $T_s^{\rm MD} = 900(50)$, 950(50) and 1000(50) K with classical molecular dynamics. Similarly, for $c_v = 1\%$ and zero pressure we find $T_s^{\rm DFT} = 1075(100)$ K and $T_s^{\rm MD} = 1100(50)$ K. Therefore, we can conclude that the employed interaction potential model appears to be physically reliable for describing defective CaF_2 systems as well, as it provides results that are consistent with first-principles methods.

Regarding Li_3OCl, we initially performed a series of both classical and *ab initio* simulations of the perfect system considering $T \neq 0$ and zero-pressure conditions. In both cases, we consistently found that the crystal remains in the normal state all the way up to melting. Likewise, we simulated Li_3OCl with a concentration of vacancies of 1% at $P = 0$ GPa. The superionic transition temperature obtained in this case with DFT methods is $T_s^{\rm DFT} = 1250(100)$ K, and with classical molecular dynamics $T_s^{\rm MD} = 1160(50)$ K. Therefore, we may conclude that the adopted Li_3OCl interaction potential model appears to be physically reliable, as it provides results that are consistent with first-principles methods.

It has been already demonstrated by others that the Vashishta-Rahman potential [4] mimics bulk AgI with great accuracy at $T \neq 0$ conditions (see, for instance, Refs. [5,6]). Keen *et al.* have observed a superionic transition under hydrostatic pressure in the rock-salt phase of bulk AgI [7]. The rock-salt structure consists of two interlaced fcc sublattices relatively displaced by $(\frac{1}{2}, \frac{1}{2}, \frac{1}{2})$ in direct coordinates (space group $Fm\overline{3}m$). We considered as a good test to check whether the Vashishta-Rahman potential could reproduce also the experimental findings by Keen *et al.* Our molecular dynamics simulations show that this is actually the case. In particular, we obtain a superionic transition temperature of 450(50) K at $P = 1$ GPa that compares very well with the experimental value $T_s^{\rm expt} = 500$ K [7]. A similar good agreement is observed also with respect to AIMD simulations undertaken at same pressure conditions (i.e., $T_s^{\rm DFT} = 435(75)$ K). Therefore, we may conclude that for present purposes the Vashishta-Rahman potential represents a physically reliable interaction model, as it provides results that are consistent with both experiments and first-principles methods.

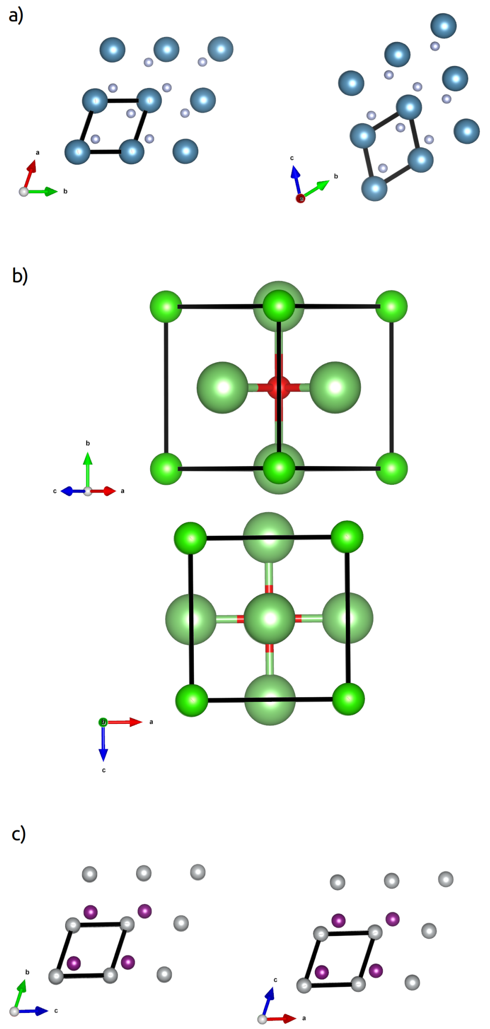

Supplementary Figure 1: Sketch of the equilibrium crystal phases of the analysed compounds considering different orientations. The corresponding primitive unit cells are marked with thick black lines. (a) CaF_2, large blue and small grey spheres represent Ca and F ions respectively; (b) Li_3OCl, large green, small green, and small red spheres represent Cl, O, and Li ions respectively; (c) AgI, white and purples spheres represent I and Ag ions respectively. In CaF_2 thin films, the equilibrium crystal structure corresponds to the cubic fluorite phase (space group $Fm\overline{3}m$) which upon biaxial tensile stresses transforms into a tetragonal phase (space group $I4/mmm$) due to the loss of cubic symmetry. In Li_3OCl thin films, that corresponds to the cubic anti-perovskite phase (space group $Pm\overline{3}m$) which upon biaxial tensile stresses transforms into a tetragonal phase (space group $P4/mmm$) also due to the loss of cubic symmetry. In AgI thin films, this corresponds to the cubic zincblende structure (space group $F\overline{4}3m$) which upon biaxial compressive stresses transforms into a tetragonal phase (space group $I\overline{4}m2$).

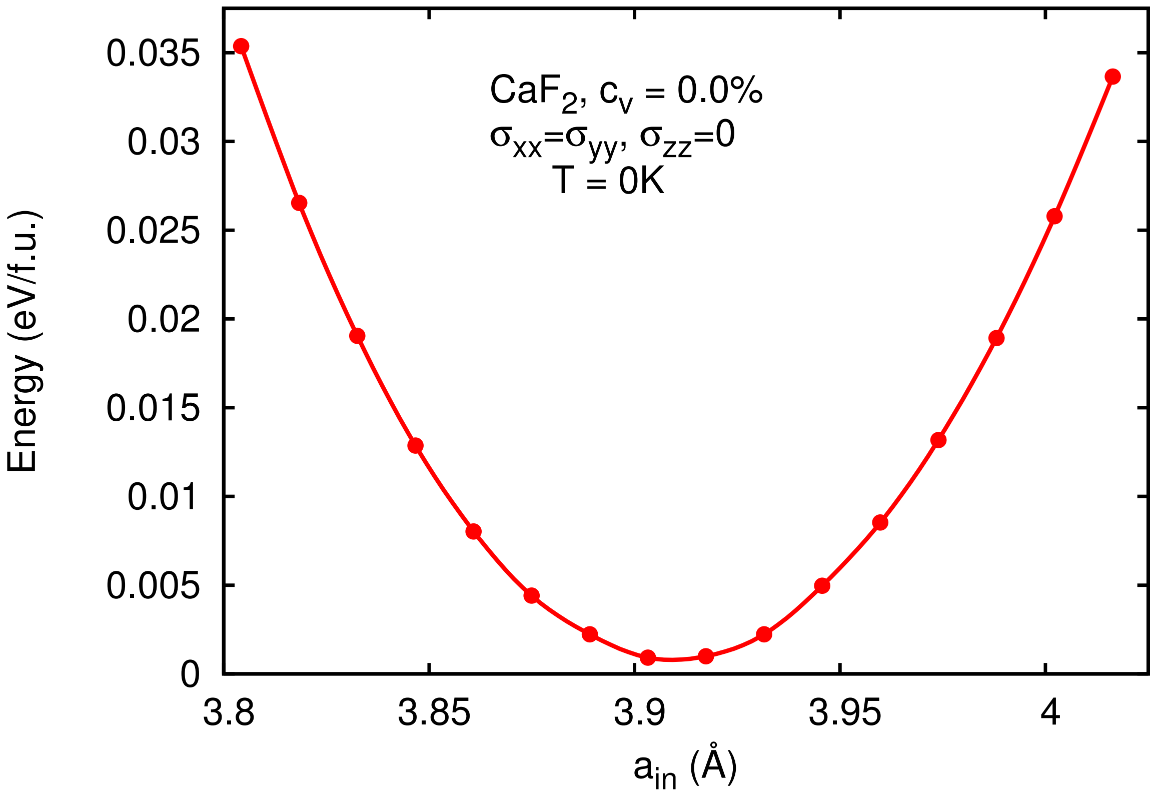

Supplementary Figure 2: Zero-temperature energy of perfect CaF_2 thin films expressed as a function of the in-plane lattice paremeter. Dots represent the energy values estimated with DFT methods and the solid line is a spline curve fitted to them. We confirm the absence of any structural phase transformation driven by strain.

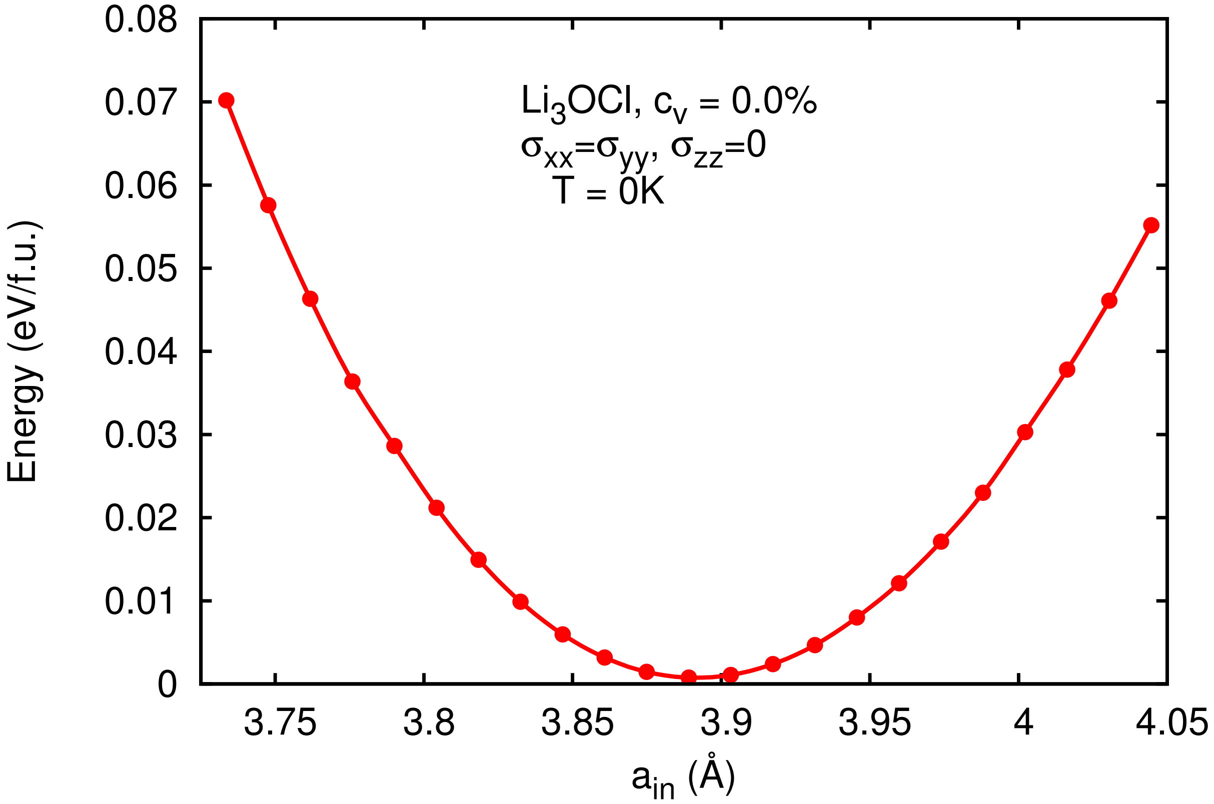

Supplementary Figure 3: Zero-temperature energy of perfect Li_3OCl thin films expressed as a function of the in-plane lattice paremeter. Dots represent the energy values estimated with DFT methods and the solid line is a spline curve fitted to them. We confirm the absence of any structural phase transformation driven by strain.

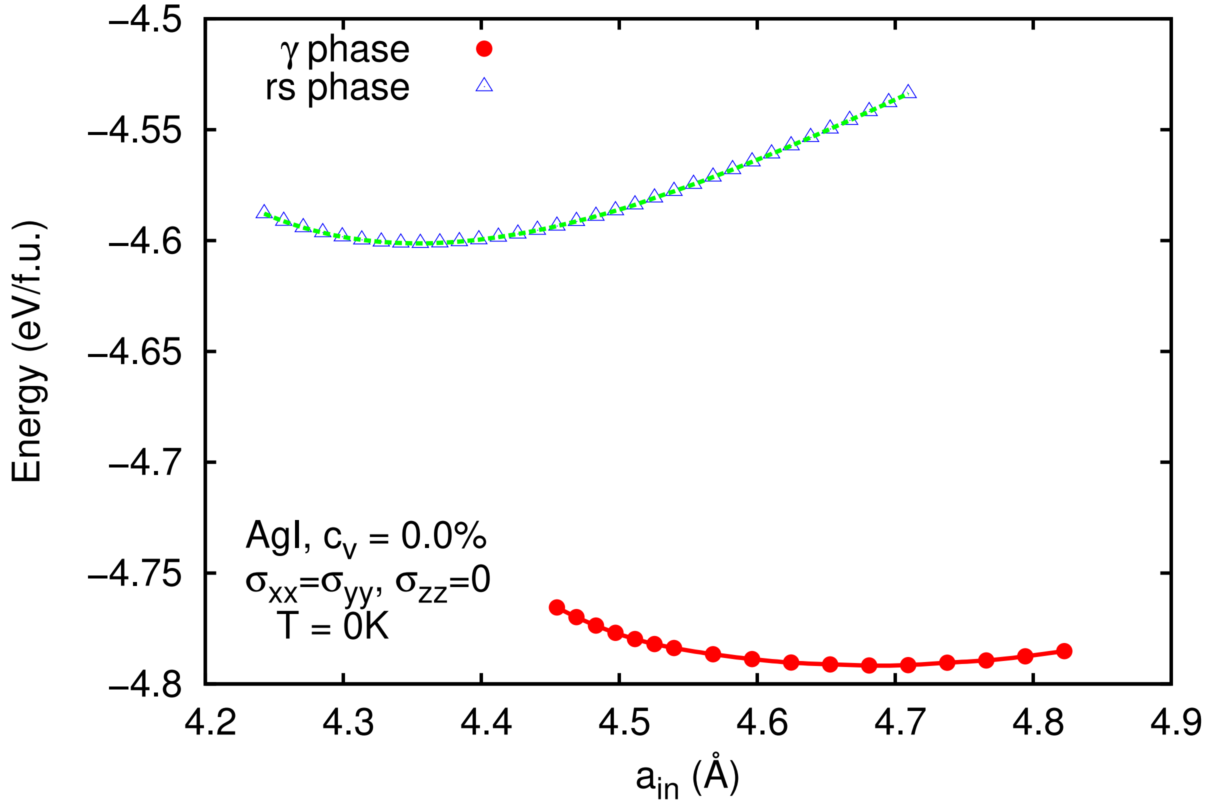

Supplementary Figure 4: Zero-temperature energy of perfect AgI thin films expressed as a function of the in-plane lattice paremeter and considering different crystal cubic phases. Dots and triangles represent the energy values estimated with DFT methods and the solid and dashed lines are spline curves fitted to them. We have considered also the cubic rock-salt (rs) structure observed in bulk at high-pressure conditions; as it can be appreciated this is energetically non-competitive with respect to the cubic zincblende (γ) phase.

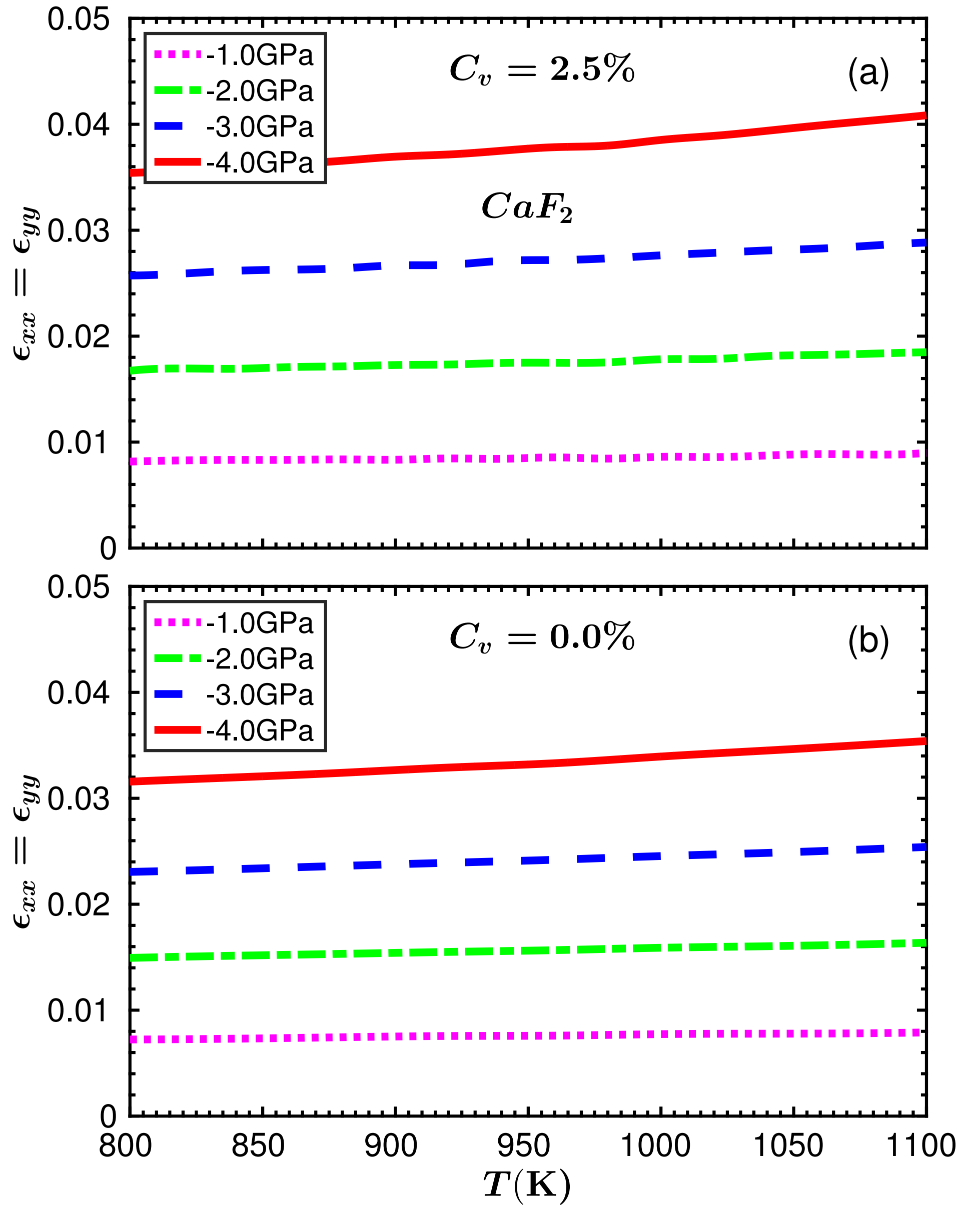

Supplementary Figure 5: In-plane strain calculated in defective (a) and perfect (b) CaF_2 thin films as a function of temperature and biaxial tensile stress. Lines represent spline curves fitted to the molecular dynamics results obtained at temperature intervals of 20 K. We find that despite the ionic F^- diffusivity in the defective system is much larger than that in the perfect system (actually, the defective system is superionic whereas the perfect remains in the normal state), the temperature variation of ϵ is very similar in both systems. Consequently, the mechanocaloric effects estimated in both cases are very similar as well.

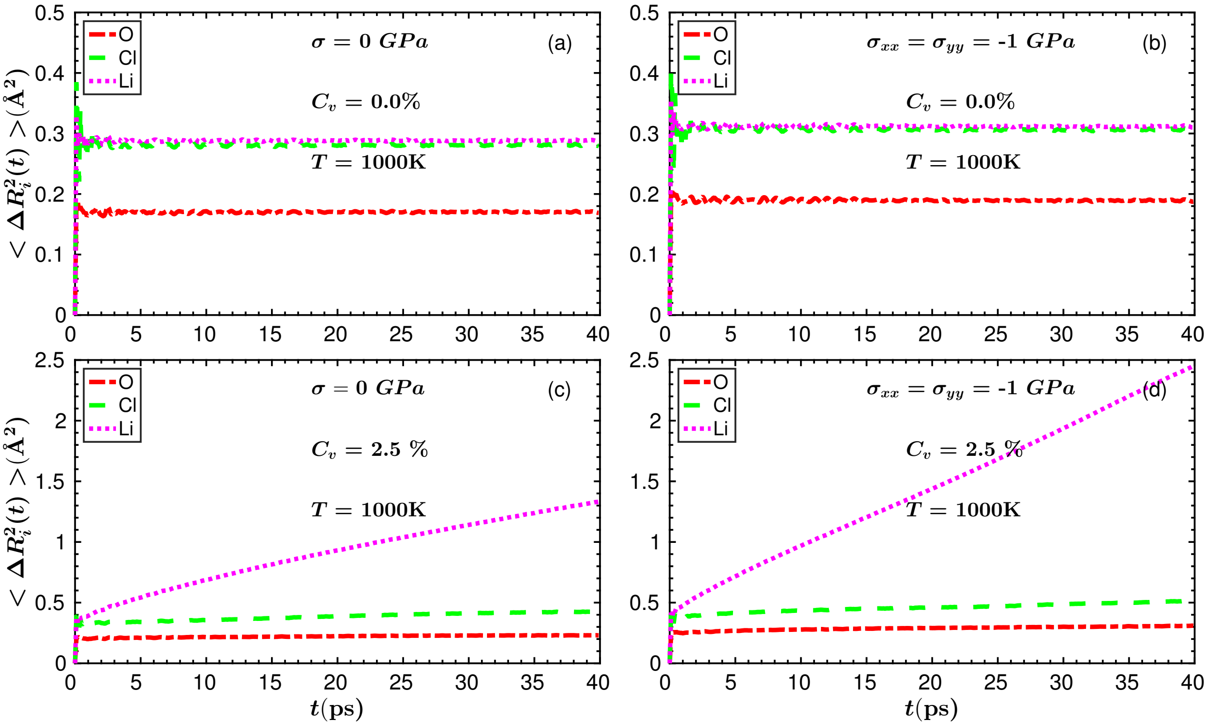

Supplementary Figure 6: Ionic mean squared displacements in Li_3OCl thin films calculated at $T = 1000$ K with molecular dynamics simulations. Results are expressed as a function of vacancy concentration and biaxial tensile stress. (a) $c_v = 0\%$ and $\sigma_{xx} = \sigma_{yy} = 0$ GPa; (b) $c_v = 0\%$ and $\sigma_{xx} = \sigma_{yy} = -1$ GPa; (c) $c_v = 2.5\%$ and $\sigma_{xx} = \sigma_{yy} = 0$ GPa; (d) $c_v = 2.5\%$ and $\sigma_{xx} = \sigma_{yy} = -1$ GPa. It is appreciated that in the absence of point defects the diffusivity of Li^+ ions is null within the considered stress-interval $0 \geq \sigma \geq -1$ GPa. In the presence of ion vacancies, however, the system becomes superionic and the Li^+ ions are highly mobile. We note that the mobility of lithium atoms is significantly enhanced under increasing tensile stress.

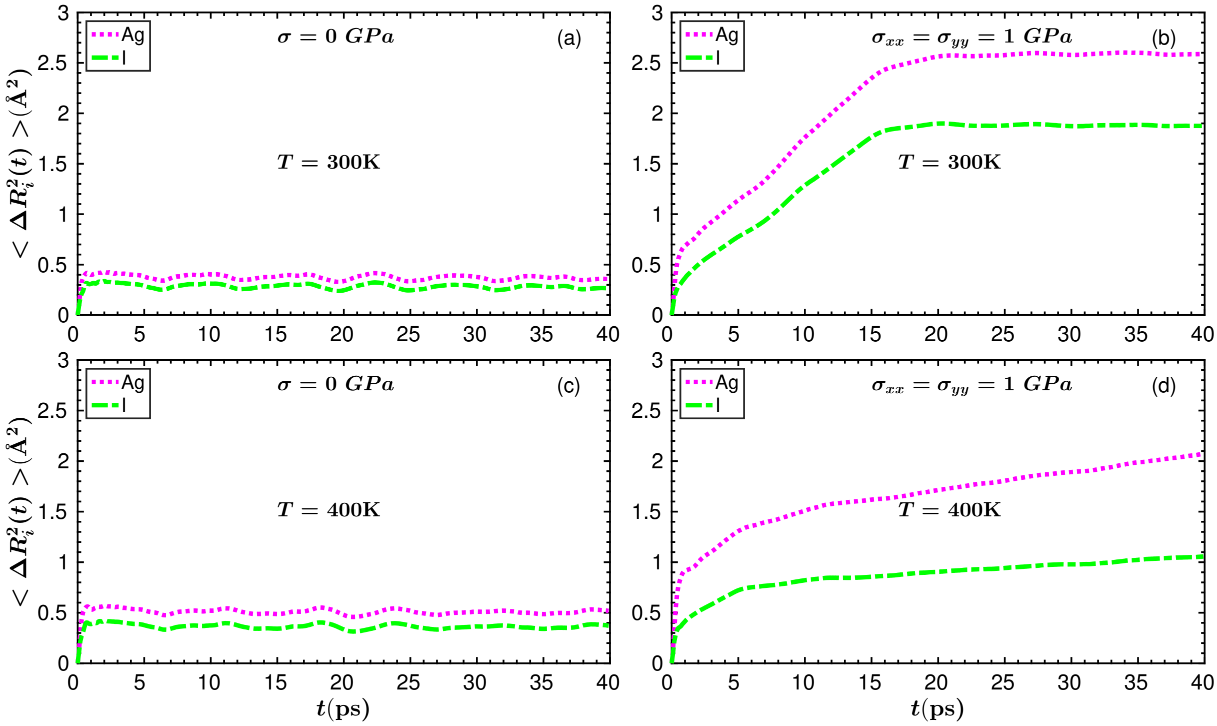

Supplementary Figure 7: Ionic mean squared displacements in perfect AgI thin films calculated with molecular dynamics simulations. Results are expressed as a function of temperature and biaxial compressive stress. (a) $T = 300$ K and $\sigma_{xx} = \sigma_{yy} = 0$ GPa; (b) $T = 300$ K and $\sigma_{xx} = \sigma_{yy} = +1$ GPa; (c) $T = 400$ K and $\sigma_{xx} = \sigma_{yy} = 0$ GPa; (d) $T = 400$ K and $\sigma_{xx} = \sigma_{yy} = +1$ GPa. In the absence of an applied biaxial stress, the system remains in the normal state both at $T = 300$ and 400 K. When a biaxial compressive stress of +1 GPa is applied, the system remains in the normal state at $T = 300$ K and becomes superionic at $T = 400$ K (i.e., the Ag^+ ions start to diffuse noticeably). We note that the ionic mean squared displacements obtained at $\sigma = +1$ GPa and $T = 300$ K are highly unusual; a certain local atomic diffusion is observed during the initial stage of the simulation, however this disappears subsequently at longer times. Actually, no real atomic diffusion occurs in the system. These features can be identified with the occurrence of a diffusionless order-disorder phase transition.

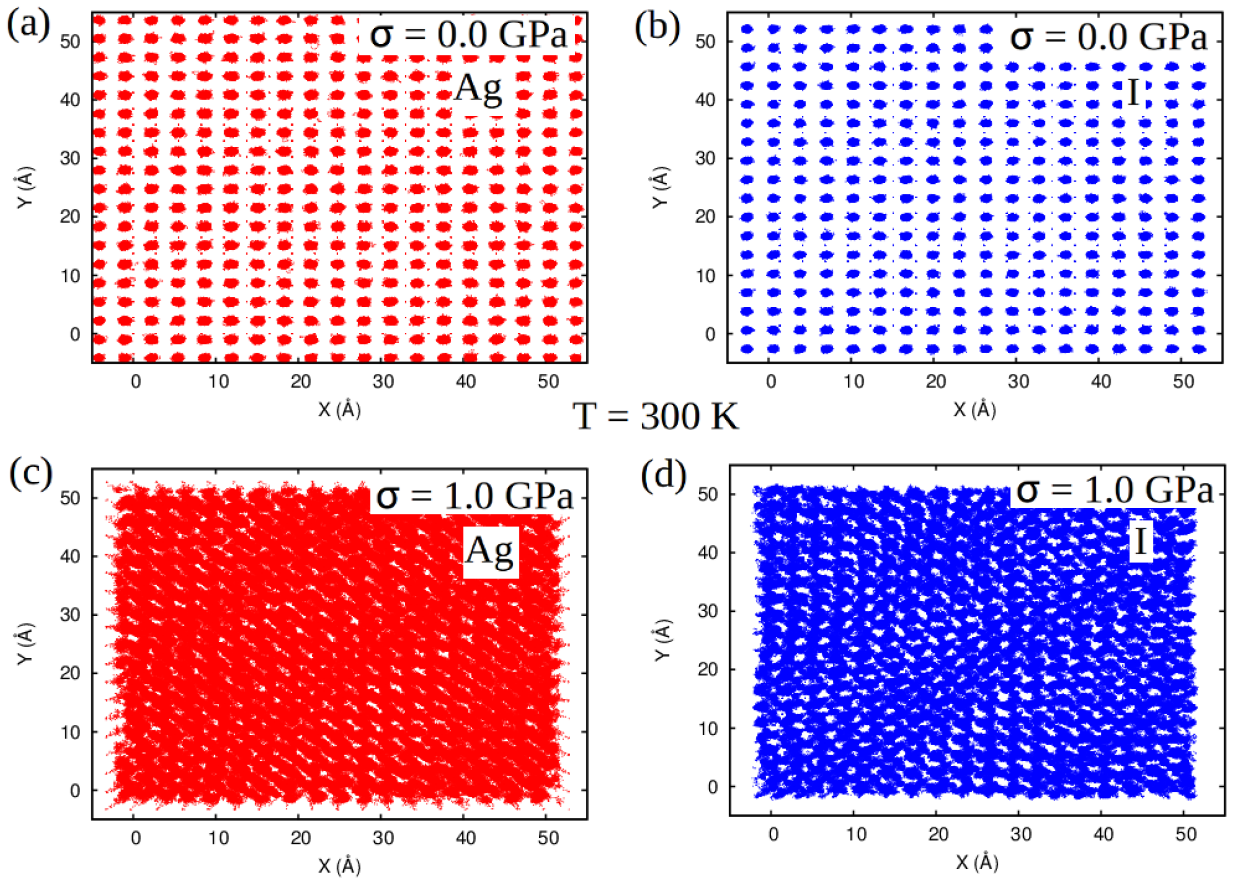

Supplementary Figure 8: Ionic density plots in perfect AgI thin films with the zincblende (γ) structure calculated with molecular dynamics simulations at $T = 300$ K. Results are expressed as a function of ionic species and biaxial compressive stress. (a) Ag and $\sigma_{xx} = \sigma_{yy} = 0$ GPa; (b) I and $\sigma_{xx} = \sigma_{yy} = 0$ GPa; (c) Ag and $\sigma_{xx} = \sigma_{yy} = +1$ GPa; (d) I and $\sigma_{xx} = \sigma_{yy} = +1$ GPa. The plots are generated by projecting the positions of the Ag^{+} and I^{-} ions, obtained during successive molecular dynamics steps in a 80 ps long simulation, over the $x - y$ plane of the simulation cell (i.e., coloured areas indicate the presence of ions in coordinate space). In the absence of an applied biaxial stress, one can easily recognise the usual fcc lattice ordering in both the cationic and anionic cases. When a biaxial compressive stress of +1 GPa is applied, however, atomic ordering is not longer appreciated although small void pockets (i.e., blank areas) are still present in the structure. These features can be identified with the stabilisation of a disordered and diffusionless phase in both the Ag^{+} and I^{-} subsystems.

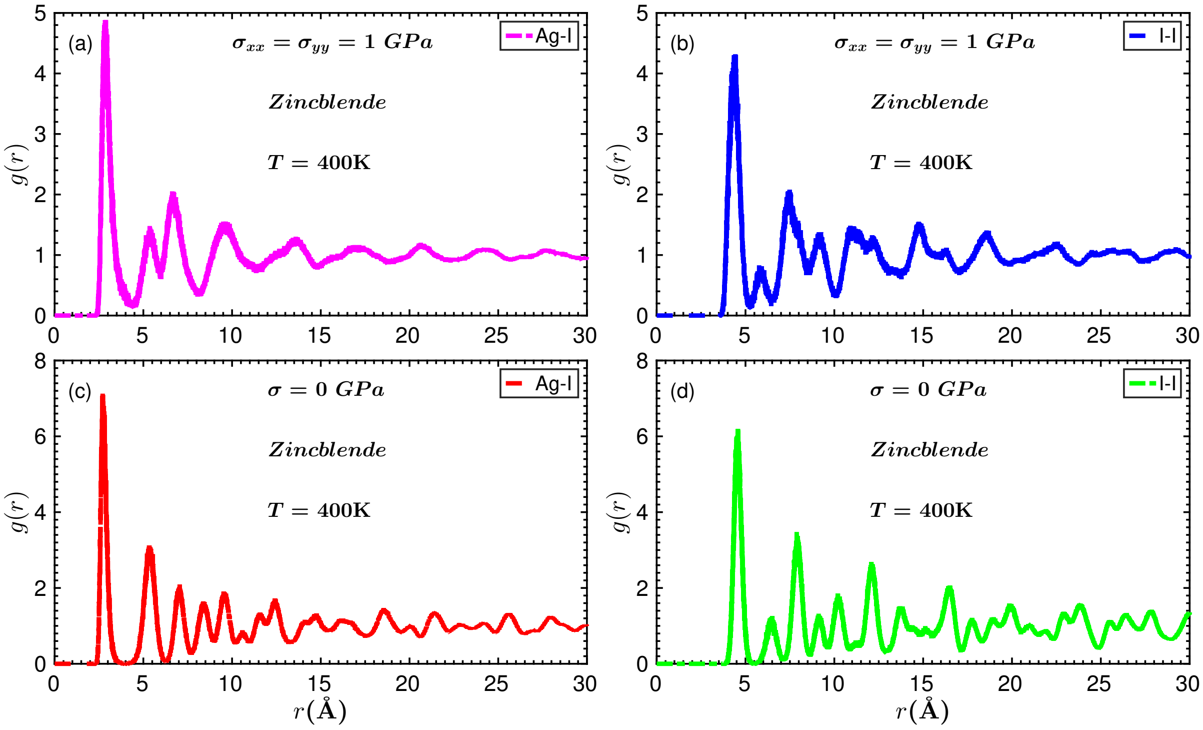

Supplementary Figure 9: Ionic radial pair distribution functions in perfect AgI thin films with the zincblende (γ) structure calculated with molecular dynamics simulations at $T = 400$ K. Results are expressed as a function of ionic pairs and biaxial compressive stress. (a) Ag-I and $\sigma_{xx} = \sigma_{yy} = +1$ GPa; (b) I-I and $\sigma_{xx} = \sigma_{yy} = +1$ GPa; (c) Ag-I and $\sigma_{xx} = \sigma_{yy} = 0$ GPa; (d) I-I and $\sigma_{xx} = \sigma_{yy} = 0$ GPa. In the absence of an applied biaxial stress the computed Ag-I and I-I curves render the typical $g(r)$ profiles obtained in crystals, in which few successive shells of neighboring atoms can be clearly identified and strong fluctuations around unity are observed at long distances. When a biaxial compressive stress of +1 GPa is applied, however, those typical solid $g(r)$ features disappear. In particular, it is not longer possible to differentiate a second shell of neighbouring atoms and the asymptotic behaviour $g(r) \sim 1$ is rapidly attained with the radial distance. In the Ag^+ lattice, we identify these traits with the presence of atomic disorder due to superionicity. In the I^- lattice, we identify these traits with the stabilisation of a disordered and diffusionless phase.

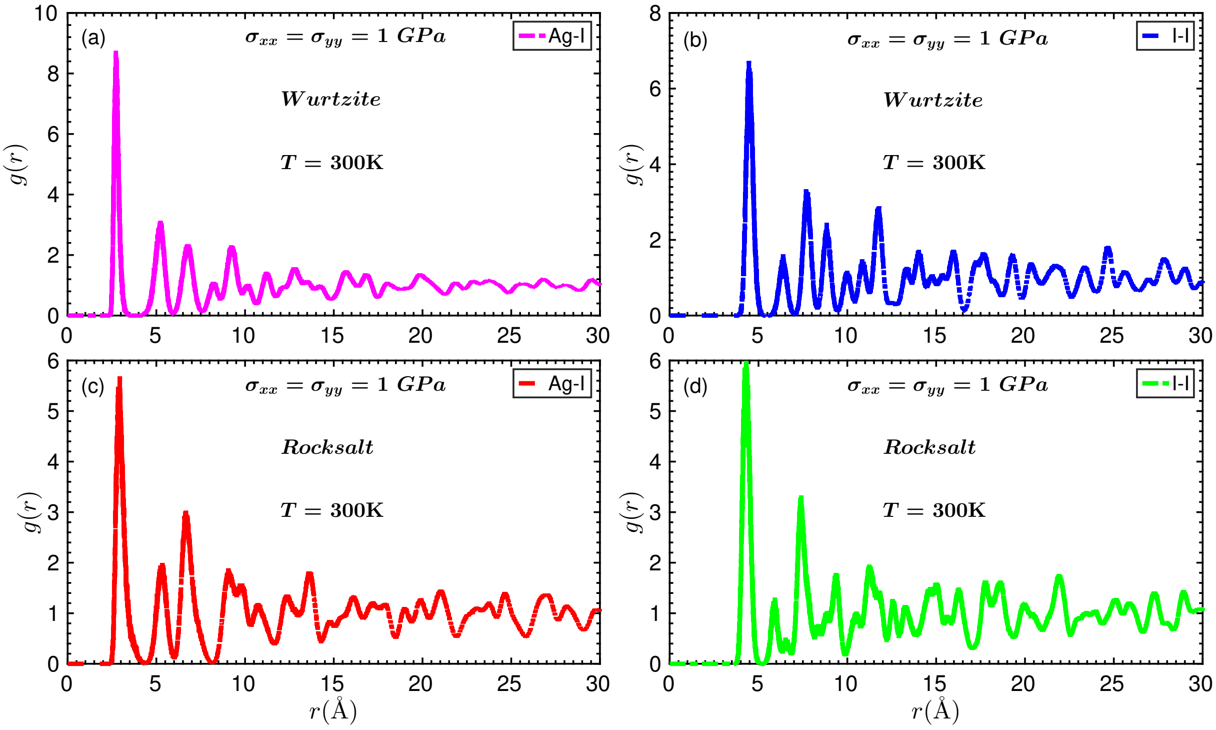

Supplementary Figure 10: Ionic radial pair distribution functions in perfect AgI thin films with the wurtzite (β) and rock-salt structures calculated with molecular dynamics simulations at $T = 300$ K and $\sigma_{xx} = \sigma_{yy} = +1$ GPa. Results are expressed as a function of ionic pairs and crystal phase. (a) Ag-I and wurtzite; (b) I-I and wurtzite; (c) Ag-I and rock-salt; (d) I-I and rock-salt. In all the cases, the computed Ag-I and I-I curves render typical $g(r)$ profiles obtained in crystals, in which few successive shells of neighbouring atoms can be clearly identified and strong fluctuations around unity are observed at long distances. There is not evidence for a σ-driven order-disorder phase transition.

SUPPLEMENTARY REFERENCES

[1] C. Cazorla and D. Errandonea, J. Phys. Chem. C **117**, 11292 (2013).

[2] C. Cazorla and D. Errandonea, Nano Letters **16**, 3124 (2016).

[3] Z. Lu, C. Chen, Z. M. Baiyee, X. Chen, C. Niu, and F. Ciucci, Phys. Chem. Chem. Phys. **17**, 32547 (2015).

[4] P. Vashishta and A. Rahman, Phys. Rev. Lett. **40**, 1337 (1978).

[5] M. Parrinello, A. Rahman, and P. Vashishta, Phys. Rev. Lett. **50**, 1073 (1983).

[6] F. Zimmer, P. Ballone, J. Maier, and M. Parrinello, J. Chem. Phys. **112**, 6416 (2000).

[7] D. A. Keen, S. Hull, W. Hayes, and N. J. G. Gardner, Phys. Rev. Lett. **77**, 4914 (1996) .
